# A Mixed-Methods Approach to Understanding Knowledge of Mosquito-Borne Infections and Barriers for Protection in Hanoi, Vietnam

**DOI:** 10.3390/tropicalmed5020066

**Published:** 2020-05-01

**Authors:** Lorraine Chapot, Thang Nguyen-Tien, Long Pham-Thanh, Hung Nguyen-Viet, Luke Craven, Johanna F Lindahl

**Affiliations:** 1 Royal Veterinary College, University of London, London NW1 0TU, UK; 2 Department of Medical Biochemistry and Microbiology, Uppsala University, SE-751 05 Uppsala, Sweden; Thang.T.Nguyen@cgiar.org (T.N.-T.); L.T.Pham@cgiar.org (L.P.-T.); johanna.lindahl@imbim.uu.se (J.F.L.); 3 International Livestock Research Institute, Ba Dinh District, Hanoi 100803, Vietnam; H.Nguyen@cgiar.org; 4 Center of Public Health and Ecosystem Research, Hanoi University of Public Health, North Từ Liêm District, Hanoi 100803, Vietnam; 5 Newcastle University Business School, Northumbria University, Newcastle-upon-Tyne NE1 4SE, UK; l.craven@unsw.edu.au; 6 Department of Clinical Sciences, Swedish University of Agricultural Sciences, SE-750 07 Uppsala, Sweden

**Keywords:** dengue, vector control, Vietnam, knowledge and practices (KPs), system network analysis

## Abstract

Dengue is a growing problem in Hanoi, with cyclical epidemics of increasing frequency and magnitude. In June 2019, we conducted a cross-sectional survey using mixed methods to investigate how inhabitants of Hanoi perceive and respond to the risk of mosquito-borne diseases (MBD). A total of 117 participants recruited using a stratified random sampling method were interviewed in three districts of Hanoi. Knowledge and practices (KP) regarding MBDs were assessed using a pre-tested questionnaire. Inferential statistics were used to identify factors associated with KP scores and describe the relationship between variables. Additionally, a “risk-mapping” exercise was conducted in a subsample through semi-structured interviews and analyzed qualitatively and quantitatively using the System Effects platform. Factors significantly associated with knowledge scores were education and family history of MBDs. While knowledge and practice scores were found to be positively correlated in the statistical analysis, this was not corroborated by our observations on the field. The results also revealed gaps in knowledge about MBDs and vectors and highlighted a general feeling of powerlessness which prevented the adoption of protective behaviors. Therefore, educational interventions which provide concrete tools to empower communities should have a positive impact on improving vector control.

## 1. Introduction

With 3.97 billion people living in at-risk areas [1], dengue is the most widespread mosquito-borne disease. Recent decades have witnessed a dramatic re-emergence of dengue fever and dengue hemorrhagic fever worldwide—with South East Asia accounting for more than 70% of cases [2], Vietnam is among the countries bearing the highest burden [3,4]. In Hanoi city, rapid urbanization and massive population growth, along with insufficient mosquito control, have contributed to the increase in dengue vector population, leading to more frequent and cyclical epidemics [5,6]. The largest recorded outbreak occurred in 2017, when 36,354 cases and 7 deaths were reported [7].

As potential vaccines are still undergoing development, dengue prevention mainly relies on vector control [5,8]. While insecticide-based methods are facing numerous issues such as increasing resistance in *Aedes sp.* or environmental toxicity, researchers have been exploring alternative approaches often focusing on environmental management. Numerous studies have highlighted the critical importance of community participation in the elimination of breeding sites and emphasized the need for information and education to promote the adoption of protective practices [9,10,11,12,13,14,15,16,17,18,19,20]. However, despite increased risk communication efforts by health authorities and NGOs, control interventions are still limited by a lack of engagement by local authorities and communities in disease prevention. Achieving behavioral changes remains a key challenge that requires a deep understanding of local knowledge and practices to design and implement effective and sustainable vector control strategies.

This study aimed to investigate risk perception and prevention practices among inhabitants of Hanoi in order to identify barriers to the adoption of vector control measures. This will help inform future educational interventions.

## 2. Material and Methods

### 2.1. Study Design

This is a cross-sectional study relying on mixed methods. It was conducted in the vector season for 6 days between 10 and 27 June 2019. It included a knowledge and practices (KPs) survey and a mapping exercise to further investigate the barriers to vector control.

### 2.2. Study Area and Sample

Hanoi is the capital and second most populous city in Vietnam, with 7.8 million inhabitants, resulting in a density of 2239 inhabitants/km^2^, and has one of the highest urbanization rates in Asia [9,21]. Hanoi city is divided into 12 urban and peri-urban districts and 18 rural districts. For the aim of this study, one urban (Ba Dinh), one peri-urban (Ha Dong) and one rural district (Chuong My) were purposely chosen. In each district, four communes were identified using randomly generated GPS points and 120 households were selected for the KP study employing a stratified random sampling method. Participants in the mapping exercise were sampled from among respondents to the KP study using a convenience sampling procedure.

### 2.3. Ethical Approval

This study received ethics approval from the Royal Veterinary College (URN SR2019-0241) and Hanoi University of Public Health (280/2019/YTCC-HD3). Informed consent was obtained from the respondents before initiation of the survey. All participants had the opportunity to withdraw themselves from the study at any point. Data from the questionnaires was anonymized using ID numbers and encrypted on the servers of the London School of Hygiene and Tropical Medicine, London.

### 2.4. KP Questionnaire

A questionnaire was developed in Open Data Kit (https://getodk.org) and pre-tested to ascertain comprehensibility. Minor revisions were made afterwards. The questionnaire was divided into three sections: the first part aimed to collect the respondent’s demographic characteristics; the second included eight items assessing knowledge about MBD causes, symptoms, risk factors, and vector biology and three items assessing usual protection practices; the third asked about family history of MBDs and sources of health information. The questionnaire was administered through face-to-face interviews. Questions about knowledge were awarded one point per correct answer and questions about practices were awarded one point per protective behavior.

### 2.5. Mapping Exercise

A mapping exercise was conducted through semi-structured interviews for system effects modelling [22]. Participants were provided a template with the focus question “Why do I get bitten by mosquitoes?”. They were asked to write down the factors they thought were related to the risk of getting bitten and indicate the connections between them by arrows. This resulted in directed graphs representing a network of factors centered on the focus question. The exercise was pre-tested using different framing of the focus question in order to assess comprehensibility and refine the formulation.

### 2.6. Statistical Analysis

Descriptive statistics were used to analyze respondents’ demographic characteristics. The nature of the data distribution was assessed using the Kolmogorov–Smirnov test. Mann–Whitney U tests, Kruskal Wallis tests and negative binomial regression were used to identify factors associated with KP scores, while Spearman’s rho was used to describe the relationship between K and P scores. Categorical variables were expressed as percentages, continuous variables as the mean ± standard deviation and discrete variables as the median ± interquartile range (IQR). A p-value < 0.05 was taken as significant for inferential statistics. Factors with a p-value > 0.1 in univariate analysis were not included in the negative binomial model. All analyses were performed using STATA 15.0.

### 2.7. System Network Analysis

Individual graphs constructed during the mapping exercise were replicated on the System Effects platform (https://systemeffects.com/#/admin) developed by Luke Craven, Northumbria University, and translated into adjacency matrices [22]. Language and concepts of the determinants were homogenized in order to condense individual matrices into one aggregated adjacency matrix which was used to generate a directed network graph in Gephi (https://gephi.org). This graph was visually explored to investigate the relationship between factors and identify potential barriers perceived by the community. Additionally, measures of centrality and ranking of determinants by in- and outdegree were used to identify those with the highest connectivity within the network. 

## 3. Results

### 3.1. Demographics

A total of 117 questionnaires were completed. Socio-demographic characteristics of the respondents are presented in Table 1. Of the 117 respondents, 57.3% were females and 43.7% were males; the mean age was 52.13 ± 13.95, ranging from 18 to 83. In total, 86.3% had at least secondary education. The most common occupation was farming (34.2%) and 22.2% were retired.

### 3.2. Assessment of Knowledge

The median score for knowledge was 7 ± 4 (med ± IQR) ranging from 0 to 18. The proportions of answers to selected questions are shown in Table 2. The majority of respondents were aware of dengue (82.1%). However, 33.3% did not know any symptoms of MBDs. Symptoms frequently mentioned included fever (61.5%), hemorrhage (45.3%) and rash (14.5%). Most respondents recognized polluted (59.8%) or stagnant water collections (62.4%) as potential mosquitoe-breeding sites but only 1.7% also mentioned clean water collections, which are preferred by *Aedes sp*. [6]. In total, 76.9% correctly identified either summer or rainy season as high-risk periods. The main source of health information was television (65.8%), followed by local public communication by loudspeaker (36.8%). In contrast, health workers (9.4%) and social media (3.4%) seemed to play a minor role in informing about MBDs. 

### 3.3. Assessment of Practices

The median score for practices was 3 ± 1 (med ± IQR), ranging from 0 to 7. The proportions of various preventive methods reported by the participants are shown in Table 3. The most commonly used were bed nets (83.8%) and insecticides (65.8%). When asked about the frequency of elimination of both natural and domestic breeding sites such as water containers, discarded tires and various wastes, 76.1% reported eliminating breeding sites at least weekly and 49.6% daily. Personal protective measures such as coils, repellents and covering clothing were rarely mentioned. 

### 3.4. Association Between KP Scores and Demographic Variables

Results of the statistical analyses are presented in Table 4. Factors with a p-value < 0.1 in univariate analysis were further assessed using binomial regression to control for confounding. Education (p-value = 0.001) and family history of MBDs (p-value = 0.04) were the two factors found to be significantly associated with knowledge scores. No factor was found to be associated with practices.

### 3.5. Correlation Between Knowledge and Practices

Spearman’s rank correlation indicated a strong positive correlation between knowledge and practices (Spearman’s rho = 0.6161, p < 0.001).

### 3.6. System Network Analysis

**Figure 1:**
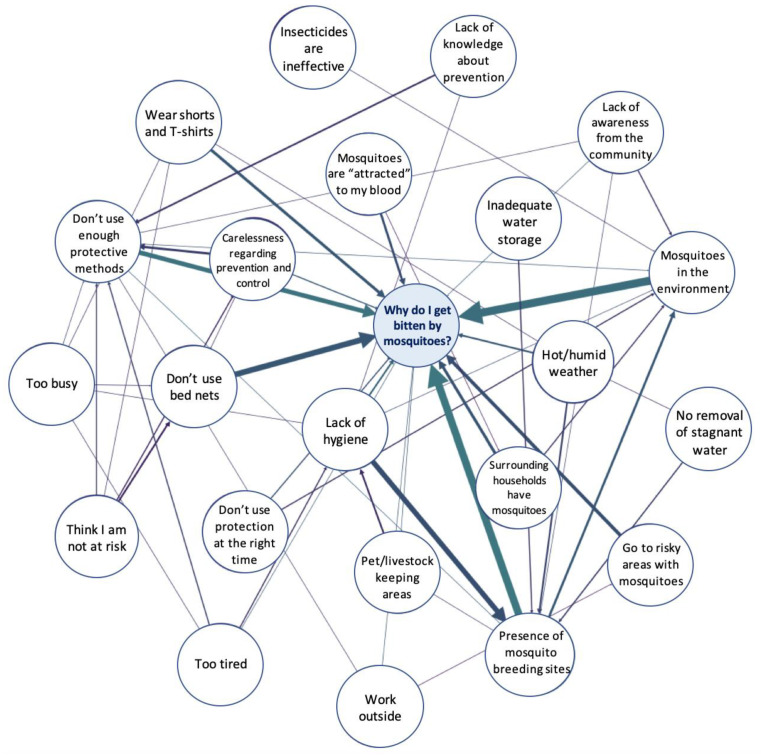
Aggregated map of determinants of mosquito control in households in Hanoi, Vietnam. The thickness of each edge is proportional to its weight, i.e., the number of participants who identified this link.

**Figure 2:**
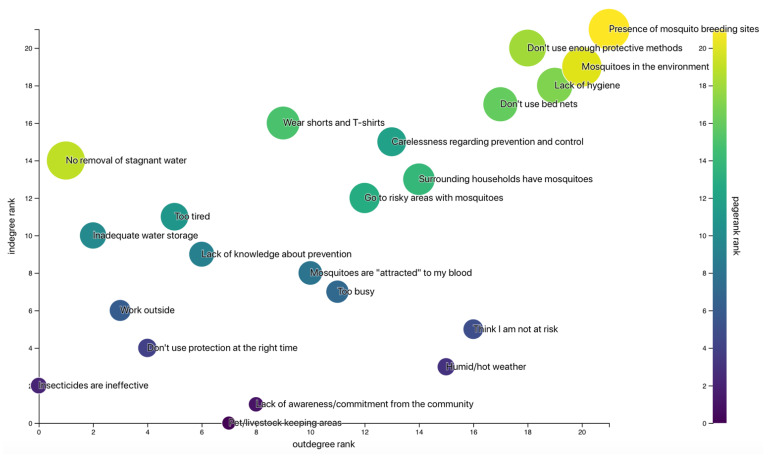
Ranking of determinants by in- and outdegree. Circles are sized according to the PageRank value of each factor.

In total, 31 individual graphs were constructed during semi-structured interviews and aggregated into one graph representing the participants’ perception of risk factors for getting bitten by mosquitoes (Figure 1). In addition to its visual exploration, degree and centrality measures presented in Table 5 and Figure 2 showed that the most influential factors identified by the participants were the presence of breeding sites in the environment, the lack of protective measures and the lack of hygiene. Factors related to the lack of protection included misuse of bed nets, poor knowledge about prevention, low risk perception and an attitude of negligence. Overall, our discussions with the participants highlighted a fatalistic attitude regarding disease control: while a majority believed they were at risk of getting infected, they perceived the presence of mosquitoes in the environment as something natural and did not think they could have control over it. Most of them did not feel responsible for eliminating breeding sites and blamed the neighboring households for not performing doing so properly. Additionally, some participants believed mosquitoes could not bite during the day and therefore neglected to use bed nets when sleeping at noontime.

## 4. Discussion

Our study highlighted gaps in knowledge of inhabitants of Hanoi about dengue disease and vectors. Although most of the surveyed population were aware of dengue, one-third of the respondents were unable to give details of any symptoms and most of the remaining respondents only mentioned fever. This is likely to contribute to underestimating the occurrence of dengue fever and result in people not seeking adequate care. Mistaken beliefs also resulted in improper protective behaviors and limited use of personal protection, with a minority of respondents being aware that mosquitoes could bite during the day and reproduce in clean water collections, which could possibly reflect a confusion with the malaria vector *Anopheles sp*. A previous study conducted in Laos in 2009 revealed that one-third of respondents believed dengue and malaria were transmitted by the same vector and could not differentiate between the two diseases [23]. This misconception could contribute to the implementation of inappropriate *Aedes* control measures. Our results also suggested limited engagement in spreading health information by health workers.

In agreement with previous studies, we found the level of education and history of MBDs to be associated with knowledge [21,24,25]. Unlike most of those studies, we also found a positive correlation between knowledge and practices. However, this was contradicted by our observations in the field: although almost half of the participants reported eliminating breeding sites daily, we were able to collect mosquito larvae on most household premises (results not shown), particularly in rural and peri-urban areas, where the presence of rubbish and tires provided multiple breeding sites. This result is therefore likely to be affected by declaration bias. The influence of knowledge on the adoption of preventive behaviors has been inconsistently described in the scientific literature, with the majority of studies failing to demonstrate any link between a community’s knowledge and better practices [25,26,27,28]. Knowledge does not always translate into more adequate behaviors, and risk perception has been shown to be a key component of the adoption of protective practices. A study in Malawi has shown for example that showing freshly captured mosquitoes to participants was more effective than educational leaflets in improving their understanding of the risk and increasing the use of bed nets [29].

The “risk-mapping” exercise allowed us to better understand the perception of risk and barriers preventing the implementation of protective measures. To our knowledge, this is the first time that system effects modelling was employed to explore the factors related to the perceived risk and prevention of mosquito bites. Overall, it highlighted a fatalistic attitude towards disease control combined with a lack of personal responsibility. The majority of participants did not believe that they have control over the risk and perceived the presence of mosquitoes in the environment as inevitable. This corroborates health-belief models, which have shown that perception of self-efficacy is a crucial component of the translation of risk perception to a concrete preventive behavior [28,30]. This can also be related to the findings of a previous study in Martinique, which interpreted the participants’ reluctance to apply dengue prevention despite a good risk perception as the result of a “normalization” process and questioned the effectiveness of risk communication for community mobilization in endemic areas [28]. In addition, most participants blamed their neighbors or health authorities for improper management of breeding sites rather than taking responsibility for it. Similar attitudes were described in a study conducted in Hanoi in 2017, where most people would rely exclusively on health authorities for implementing vector control [31].

Our study had several limitations. Firstly, all interviews were conducted during the day when most workers were absent, which can explain the high proportion of retired individuals and limit the representativity of the sample. Prevention practices were self-reported and therefore declaration bias cannot be ruled out, as demonstrated by our contradictory observations on site. Selection of participants for the “mapping exercise” relied mostly on convenience sampling. The exercise would also have benefited from a preparatory training session to give participants more autonomy and limit the influence of the interviewer when constructing the graphs.

## 5. Conclusion and Recommendations

Our study demonstrates the existence of barriers to implementing vector control measures in Hanoi, which included improper knowledge of vector biology and disease features, low self-responsibility and low perceived self-efficacy. In order for risk awareness to translate into actual protective behaviors, we recommend the adoption of participatory approaches to engage communities and encourage compliance by demonstrating the feasibility and effectiveness of vector control measures, in coordination with local authorities.

## Figures and Tables

**Table 1:** Socio-demographic profile of participants.

Characteristic	% (N = 117)
**Gender**	
Male	42.7 (50)
Female	57.3 (67)
**Age (Mean ± SD)**	52.13 ± 13.95
18–39	20.5 (24)
40–49	23.9 (28)
50–59	24.8 (29)
60+	30.8 (36)
**Education**	
≤Primary school	13.7 (16)
Secondary school	34.2 (40)
High school	34.2 (40)
≥College/University	17.9 (21)
**Occupation**	
Unemployed	4.3 (5)
Farmer	34.2 (40)
Worker/Seller	17.1 (20)
Public or private services	11.1 (13)
Self-employed	9.4 (11)
Retired	22,2 (26)
Student	1.7 (2)
**Family Member Diagnosed with Dengue**	
Yes	9.4 (11)
No	90.6 (106)

**Table 2:** Proportion of responses to various questions about knowledge.

Question	% (N = 117)
**Which Disease(s) Transmitted by Mosquitoes Have you Heard About?**	
Don’t know any	12.8 (15)
Dengue	82.1 (96)
Japanese encephalitis	0.9 (1)
Zika	1.7 (2)
Malaria	15.4 (18)
What Symptoms of MBDs do You Know? *	
Don’t know any	33.3 (39)
High fever	61.5 (72)
Muscle pain	4.3 (5)
Nausea/vomiting	2.6 (3)
Severe headache	4.3 (5)
Rash	14.5 (17)
Hemorrhage	45.3 (53)
What do You Think Can Increase the Risk of Getting Infected with MBDs? *	
Don’t know	46.2 (54)
Warm and wet season	36.8 (43)
High population density	1.7 (2)
Stagnant water	28.2 (33)
Livestock keeping	16.2 (19)
Can You List Mosquito Breeding Sites? *	
Don’t know any	6.0 (7)
Clean water collection	1.7 (2)
Drain/polluted water	59.8 (70)
Stagnant water containers	62.4 (73)
Tires	5.1 (6)
Water tanks, jars or buckets	25.6 (30)
Vase	6.0 (7)
Bonsai rockery	6.0 (7)
Garbage/rubbish	2.6 (3)
**Which Season do You Think is Most at Risk for MBDs?**	
Spring	18.8 (22)
Summer	51.3 (60)
Autumn	2.6 (3)
Winter	0.9 (1)
No difference	0.9 (1)
Rainy season	25.6 (30)

* more than one answer can be reported.

**Table 3:** Proportion of responses to various questions about practices.

Question	% (N = 117)
Which Methods do You Use to Prevent Yourself and Your Family from Getting Infected with MBDs? *	
Don’t use any	0.9 (1)
Screening of doors/windows	0.9 (1)
Mosquito repellent creams/liquid	16.2 (19)
Mosquito nets	83.8 (98)
Electric rackets	35.9 (42)
Mosquito coils/Incense sticks	2.6 (3)
Covering clothes	3.4 (4)
Keeping lids on water tanks	3.4 (4)
Use of chemicals in water containers	4.3 (5)
Anti-mosquito products (e.g., insecticides)	65.8 (77)
Elimination of breeding sites	44.4 (52)
Fish in water containers	6.8 (8)
Mosquito traps inside home	12.8 (15)
**How Often do You Remove Mosquito Breeding Sites?**	
Never	0.9 (1)
Once in several months	11.1 (13)
Once per month	1.7 (2)
2–3 times per month	6.8 (8)
Once a week	6.8 (8)
2–3 times per week	19.7 (23)
Daily	49.6 (58)
Only after raining	3.4 (4)

* more than one answer can be reported.

**Table 4:** Knowledge (K) and practice (P) scores with respect to demographics.

Variable	K Score 7 ± 4	p-Value (Univariable)	p-Value (Multivariable)	P Score 3 ± 1	p-Value (Univariable)	p-Value (Multivariable)
Gender *						
Male	8 ± 3	0.138	-	4 ± 3	0.610	-
Female	7 ± 5			3 ± 2		
Age **						
18–39	7 ± 5.5	0.429	-	3 ± 2	0.697	-
40–49	7 ± 3.5			3 ± 2		
50–59	8 ± 6			3 ± 2		
60+	7.5 ± 5			3.5 ± 2		
District **						
Chuong My	7 ± 4	0.065	0.092	3 ± 2	0.019	0.142
Ha Dong	8 ± 5			4 ± 2		
Ba Dinh	8 ± 3			4 ± 2		
Education **						
≤Primary school	6 ± 5	0.003	0.001	3 ± 2	0.041	0.233
Secondary school	7 ± 3.5			3 ± 2.5		
High school	8.5 ± 4			4 ± 2		
≥ College/University	8 ± 4			3 ± 2		
Occupation**						
Unemployed	6 ± 1	0.022	0.396	3 ± 2	0.003	0.319
Farmer	7 ± 4.5			3 ± 2		
Worker/Seller	7 ± 2.5			3 ± 0.5		
Public or private services	9 ± 5			5 ± 2		
Self-employed	8 ± 6			5 ± 3		
Retired	9 ± 5			4 ± 2		
Student	3.5 ± 1			2 ± 4		
Family member diagnosed with dengue *						
Yes	12 ± 6	0.047	0.050	4 ± 1	0.032	0.188
No	7 ± 0.4			3 ± 1		

* Mann–Whitney U test; ** Kruskal–Wallis test; Note: Data are presented as the median ± interquartile range.

**Table 5:** Network statistics of key determinants.

Factor	Weighted Indegree	Weighted Outdegree	Eigencentrality	PageRank
Presence of mosquito breeding sites	20	17	0.525666	0.098047
Do not use enough protective methods	14	10	0.065081	0.064626
Lack of hygiene	7	13	0.035786	0.043412
Do not use bed nets	5	10	0.039116	0.032595
